# ALIBY: ALFA Nanobody-Based Toolkit for Imaging and Biochemistry in Yeast

**DOI:** 10.1128/msphere.00333-22

**Published:** 2022-10-03

**Authors:** Dipayan Akhuli, Anubhav Dhar, Aileen Sara Viji, Bindu Bhojappa, Saravanan Palani

**Affiliations:** a Department of Biochemistry, Division of Biological Sciences, Indian Institute of Science, Bangalore, India; University of Georgia

**Keywords:** *Saccharomyces cerevisiae*, biochemistry, cell biology, cell division, fluorescence, fluorescent image analysis, live-cell imaging, mitochondria, nanobody, vacuoles

## Abstract

Specialized epitope tags continue to be integral components of various biochemical and cell biological applications such as fluorescence microscopy, immunoblotting, immunoprecipitation, and protein purification. However, until recently, no single tag could offer this complete set of functionalities on its own. Here, we present a plasmid-based toolkit named ALIBY (ALFA toolkit for imaging and biochemistry in yeast) that provides a universal workflow to adopt the versatile ALFA tag/^Nb^ALFA system within the well-established model organism Saccharomyces cerevisiae. The kit comprises tagging plasmids for labeling a protein of interest with the ALFA tag and detection plasmids encoding fluorescent-protein-tagged ^Nb^ALFA for live-cell imaging purposes. We demonstrate the suitability of ALIBY for visualizing the spatiotemporal localization of yeast proteins (i.e., the cytoskeleton, nucleus, centrosome, mitochondria, vacuole, endoplasmic reticulum, exocyst, and divisome) in live cells. Our approach has yielded an excellent signal-to-noise ratio without off-target effects or any effect on cell growth. In summary, our yeast-specific toolkit aims to simplify and further advance the live-cell imaging of differentially abundant yeast proteins while also being suitable for biochemical applications.

**IMPORTANCE** In yeast research, conventional fluorescent protein tags and small epitope tags are widely used to study the spatiotemporal dynamics and activity of proteins. Although proven to be efficient, these tags lack the versatility for use across different cell biological and biochemical studies of a given protein of interest. Therefore, there is an urgent need for a unified platform for visualization and biochemical and functional analyses of proteins of interest in yeast. Here, we have engineered ALIBY, a plasmid-based toolkit that expands the benefits of the recently developed ALFA tag/^Nb^ALFA system to studies in the well-established model organism Saccharomyces cerevisiae. We demonstrate that ALIBY provides a simple and versatile strain construction workflow for long-duration live-cell imaging and biochemical applications in yeast.

## INTRODUCTION

Saccharomyces cerevisiae is a well-established model system for studying basic principles of eukaryotic cell biology. It is a simple, unicellular eukaryote that permits easy genetic modifications such as gene deletions or tagging using a standard PCR-based strategy (1). Numerous epitope tags have been designed to aid in investigating protein localization and performing biochemical analysis. Maltose binding protein (MBP) and glutathione *S*-transferase (GST) tags have been used for the affinity purification of fusion proteins but can impose a heavy metabolic load when overexpressed (2). FLAG, hemagglutinin (HA), and myc tags are utilized for immunostaining, immunoprecipitation, immunoblotting, and protein purification experiments, but their large antibody partners make them unsuitable for superresolution microscopy (3). The HA tag has been reported to lose its immunoreactivity during apoptosis, limiting its use in cell death-related studies (4). SPOT and EPEA tags perform well in superresolution microscopy, but their nanobody (camelid monomeric single-domain antibody [sdAb]) partners show nonspecific binding and have low binding affinities (5–7). Additionally, live-cell imaging has not been demonstrated for these tags (3). A cause for concern is the variability in the dynamics of any given protein tagged with different epitope tags for different purposes. The field of yeast cell biology currently lacks a universal tag that can cover a wide range of biological applications.

The recently developed ALFA tag excels at all of these functionalities simultaneously (3) and has been successfully employed in bacterial and mammalian systems (8–12). Here, we aim to extend the ALFA/^Nb^ALFA system to the model organism Saccharomyces cerevisiae via a plasmid-based toolkit named ALIBY (ALFA toolkit for imaging and biochemistry in yeast). The kit consists of two sets of plasmids: (i) tagging plasmids for labeling a protein of interest (POI) at its native genomic locus with a C-terminal ALFA tag and (ii) detection plasmids expressing ^Nb^ALFA, the nanobody that specifically recognizes the ALFA tag, which is tagged to a fluorescent protein under the control of various promoters for the *in vivo* detection and visualization of the ALFA-tagged POI (POI^ALFA^). ALIBY can streamline the process of yeast strain construction for diverse biochemistry and cell biology studies, making it less laborious and expensive. To this end, we demonstrate the functionality of ALIBY for live-cell imaging, immunofluorescence (IF), immunoblotting, and immunoprecipitation of various cytoskeletal and other proteins in Saccharomyces cerevisiae.

## RESULTS

### Design of plasmids.

**FIG 1:**
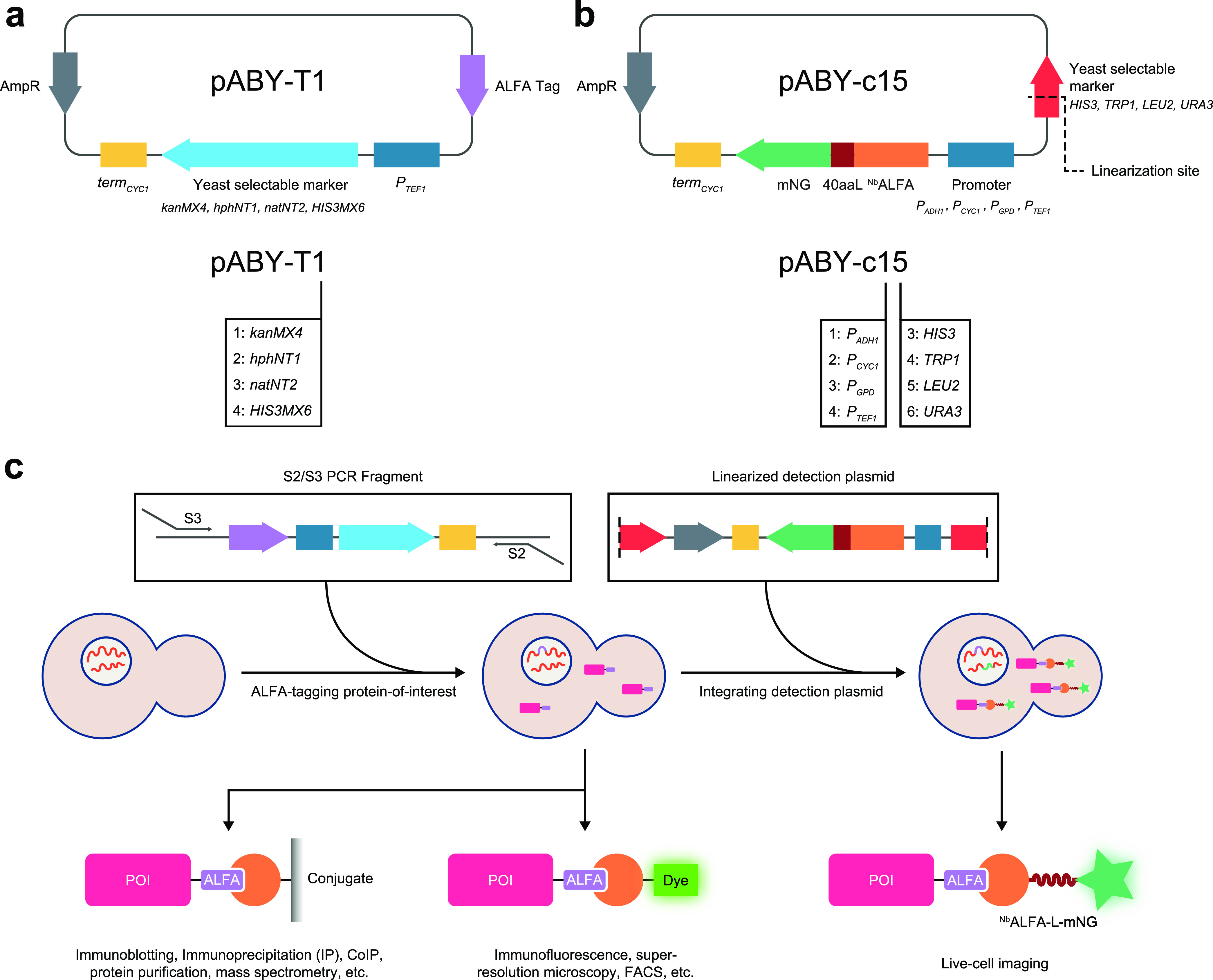
Design of detection and tagging plasmids comprising the toolkit and the proposed workflow. (a) Schematic map of a representative tagging plasmid (top) and its naming scheme (bottom). The ALFA tag amino acid sequence is (P)SRLEEELRRRLTE. The number in the plasmid name code corresponds to the yeast selectable marker, as indicated. (b) Schematic map of a representative pRS-based detection plasmid (top) and its naming scheme (bottom). 40aaL, 40-amino-acid linker; mNG, mNeonGreen. AmpR encodes a β-lactamase responsible for conferring resistance to ampicillin, used as a bacterial selectable marker. The black dashed line marks the linearization site in the yeast selectable marker gene. In the plasmid name code, the first digit corresponds to the promoter, while the second digit corresponds to the yeast selectable marker, as indicated. (c) Schematic of the recommended workflow for strain construction. The S2 primer overhang sequence is 5′-ATCGATGAATTCGAGCTCG-3′; the S3 primer overhang sequence is 5′-CGTACGCTGCAGGTCGAC-3′. Starting from a particular strain of choice (middle left), the PCR fragment generated from the tagging plasmid using S2/S3 primers (top left) can be transformed to generate the tagged strain (middle center). The linearized detection plasmid (top right) containing a promoter and the selectable marker of choice can now be integrated into the tagged strain to obtain the nanobody-containing tagged strain (middle right), which can be used for live-cell imaging (bottom right). The colored blocks in the top row correspond to those already labeled in panels a and b. The color-code for schematics in the bottom row is as follows: pink, POI; purple, ALFA tag; orange, ^Nb^ALFA; fluorescent green, dye; brown, 40-amino-acid linker; green, mNeonGreen. FACS, fluorescence-activated cell sorting.

We engineered two families of plasmids as part of ALIBY: (i) tagging plasmids and (ii) detection plasmids. The tagging plasmids are based on the pYM series of plasmids (13) and serve as the template for the amplification of the ALFA tag-containing PCR cassette using the universal S2/S3 primer set (1, 13) (Fig. 1a and Table 1). This PCR cassette, containing one of four selectable markers of choice (*kanMX4*, *hphNT1*, *natNT2*, and *HIS3MX6*), can be used to fuse the ALFA tag to the C terminus of any POI via a commonly used homologous recombination-based strategy (1). The detection plasmids are based on the pRS30 series of yeast integrative plasmids (Yip) (14), and they express ^Nb^ALFA linked to mNeonGreen via a flexible 40-amino-acid linker (40aaL) at its C terminus (Fig. 1b and Table 1). mNeonGreen was selected because it is brighter, has a higher quantum yield, is more photostable, and has a lower maturation time than commonly used green and yellow fluorescent proteins (15). The 40-amino-acid linker has been used as a spacer between proteins and their fused fluorescent tags such that they do not interfere with the folding and normal functioning of the POI (16). The ^Nb^ALFA-linker-mNeonGreen construct (referred to as ^Nb^ALFA-L-mNG here) has been designed with the choice of 4 different constitutive promoters, *P_CYC1_*, *P_ADH1_*, *P_TEF1_*, and *P_GPD_* (in order of increasing promoter strength [17]), in combination with 4 different vector backbones of the pRS30 series containing *HIS3*, *TRP1*, *LEU2*, and *URA3* selectable markers (Table 1). Thus, to construct a strain with a POI C-terminally fused to the ALFA tag, a PCR cassette is generated using a tagging plasmid as the template with gene-specific S2/S3 primers, which is then transformed into the starting strain of choice. This strain can be used for biochemical purposes like immunoblotting, immunoprecipitation, coimmunoprecipitation, protein purification, mass spectrometry, and also fixed-cell imaging using immunofluorescence and superresolution microscopy. Additionally, a linearized detection plasmid containing the marker of choice and expressing the ^Nb^ALFA-L-mNG construct under the control of a suitable promoter, depending on the experimental design, can be integrated into the above-described strain carrying the ALFA tag. This strain can now be used for live-cell imaging purposes (Fig. 1c). The above-mentioned workflow significantly simplifies the strain construction process by saving time and resources and making it less laborious and extensive.

**TABLE 1:** Details of tagging and detection plasmids

Plasmid	Description	Name	Backbone	Promoter	Selectable marker	Linearization site	PCR fragment size (bp)
piSP532	pYM14-ALFA-*kanMX4*	pABY-T1	pFA6a		*kanMX4*		1,521
piSP534	pYM16-ALFA-*hphNT1*	pABY-T2	pFA6a		*hphNT1*		1,750
piSP536	pYM17-ALFA-*natNT2*	pABY-T3	pFA6a		*natNT2*		1,367
piSP538	pFA6a-ALFA-*HIS3MX6*	pABY-T4	pFA6a		*HIS3MX6*		1,365
piSP551	pRS305-*P_ADH1_*-^Nb^ALFA-L-mNG-*term_CYC1_*	pABY-c15	pRS305	*P_ADH1_*	*LEU2*	KasI	
piSP553	pRS305-*P_CYC1_*-^Nb^ALFA-L-mNG-*term_CYC1_*	pABY-c25	pRS305	*P_CYC1_*	*LEU2*	KasI	
piSP555	pRS305-*P_GPD_*-^Nb^ALFA-L-mNG-*term_CYC1_*	pABY-c35	pRS305	*P_GPD_*	*LEU2*	KasI	
piSP557	pRS305-*P_TEF1_*-^Nb^ALFA-L-mNG-*term_CYC1_*	pABY-c45	pRS305	*P_TEF1_*	*LEU2*	KasI	
piSP765	pRS303-*P_TEF1_*-^Nb^ALFA-L-mNG-*term_CYC1_*	pABY-c43	pRS303	*P_TEF1_*	*HIS3*	PstI	
piSP767	pRS304-*P_TEF1_*-^Nb^ALFA-L-mNG-*term_CYC1_*	pABY-c44	pRS304	*P_TEF1_*	*TRP1*	SnaBI	
piSP565	pRS306-*P_TEF1_*-^Nb^ALFA-L-mNG-*term_CYC1_*	pABY-c46	pRS306	*P_TEF1_*	*URA3*	NdeI	

### Characterization of plasmids.

**FIG 2:**
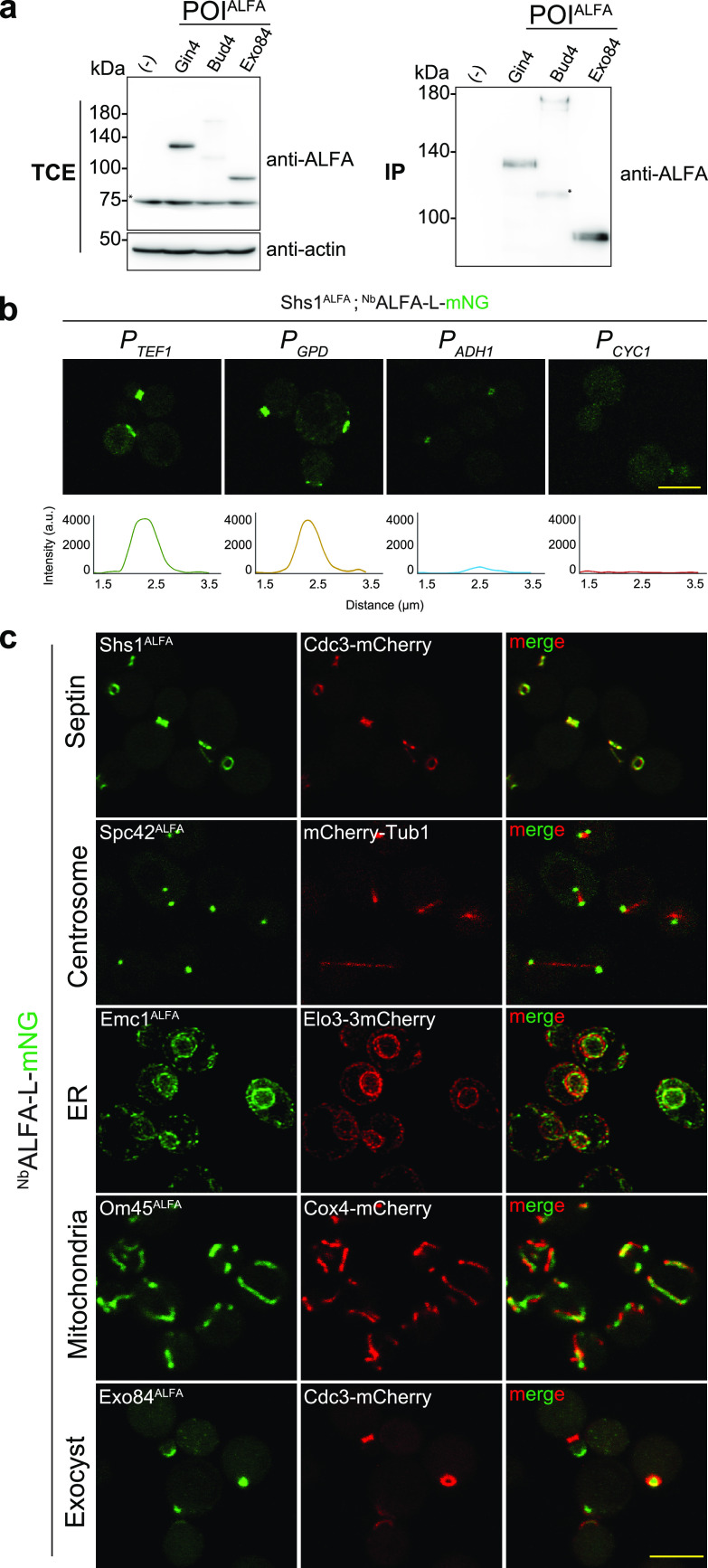
Characterization of plasmids, immunoprecipitation, and live-cell imaging of various proteins. (a) Immunoprecipitation (IP) of Gin4^ALFA^, Bud4^ALFA^, and Exo84^ALFA^. The wild-type control strain lacks the ALFA tag. Total cell extracts (TCE) of each strain were incubated with ALFA Selector^ST^ resin and washed; 1/50 of the input fraction and the entire eluate fraction were resolved by SDS-PAGE and analyzed by immunoblotting using anti-ALFA sdAb and antiactin (*n* = 3) (* indicates an unspecific band). (b) Comparison of promoters for optimizing ^Nb^ALFA expression by laser point scanning confocal microscopy. (Top row) ALFA-tagged Shs1 in ESM356 cells carrying pRS305-promoter-^Nb^ALFA-L-mNG-*term_CYC1_*. Shown from left to right are *P_TEF1_*, *P_GPD_*, *P_ADH1_*, and *P_CYC1_*. Images were captured at 1% laser excitation power at 488 nm. (Bottom row) Intensity profiles for cells with Shs1^ALFA^ expressing ^Nb^ALFA-L-mNG under the control of different promoters in the same order as the one described above. Bar, 5 μm. a.u., arbitrary units. (c) Images of ALFA-tagged proteins. From left to right (in each row) are the ALFA-tagged POI along with ^Nb^ALFA-L-mNG under the control of *P_TEF1_* (green), the mCherry-tagged marker protein (red), and the merged image. First row, Shs1, a terminal septin, with Cdc3-mCherry as a bud neck marker; second row, Spc42, a spindle pole body component, with mCherry-Tub1 as a microtubule marker; third row, Emc1, an ER membrane protein, with Elo3mCherry as an ER marker; fourth row, Om45, a mitochondrial outer membrane protein, with Cox4-mCherry as a mitochondrial marker; fifth row, Exo84, an exocyst complex component, with Cdc3-mCherry as a bud neck marker. All of the strains were grown in synthetic complete (SC) medium at 30°C. All of the images were maximum-intensity projected and deconvolved for representation. Bar, 5 μm.

To characterize the two sets of plasmids, we first constructed a set of strains, each carrying a C-terminally ALFA-tagged POI. To rigorously test the live-cell imaging capabilities of ALIBY, we chose proteins with diverse spatiotemporal dynamics and different cellular functions. Tagging was confirmed by immunoblot analysis using a horseradish peroxidase (HRP)-conjugated anti-ALFA nanobody (sdAb) (see Fig. S1a in the supplemental material). Furthermore, we performed immunoprecipitation for Gin4^ALFA^ (a septin-associated polarity kinase [18]), Bud4^ALFA^ (an anillin-like bud site selection protein [19]), and Exo84^ALFA^ (a component of the exocyst complex [20]) using the ALFA Selector^ST^ resin (Fig. 2a) and were successful in detecting a specific band corresponding to their molecular weights. This demonstrates that the tagging plasmids can be used to successfully tag yeast proteins at their C termini for biochemical applications. Furthermore, we also performed immunofluorescence analysis for ALFA-tagged Gin4, Bud4, and Exo84 using FluoTag-X2 anti-ALFA^Alexa Fluor 647^ and observed specific signals at their corresponding localization sites (Fig. S1b). These results suggest that the ALIBY toolkit can be used for both immunoblotting and immunofluorescence applications in yeast.

10.1128/msphere.00333-22.1 FIG S1 Characterization of plasmids, immunoblotting, and live-cell and immunofluorescence imaging of various proteins. (a) Confirmation of ALFA-tagged proteins by immunoblotting. Total cell extracts of the control strain lacking the ALFA tag and strains containing ALFA-tagged Emc1, Shs1, and Cdc14 were treated with HU-DTT; resolved by SDS-PAGE; and analyzed by immunoblotting using anti-ALFA HRP-conjugated sdAb and antitubulin (*n* = 3) (* indicates an unspecific band). (b) Fixed-cell immunofluorescence imaging of ALFA-tagged proteins. From left to right (in each row) are the ALFA-tagged POI stained by Alexa Fluor 647-conjugated ^Nb^ALFA (cyan), mCherry-tagged Cdc3 serving as a bud neck marker (red), and the merged image. First row, control strain lacking the ALFA tag; second row, Gin4, a protein kinase localizing to the bud neck; third row, Bud4; fourth row, Exo84. Bar, 5 μm. (c) Comparison of the intensity profiles of line scans performed for the four promoters. (d) Laser point scanning confocal microscopy for further characterization. Control strains included (i) ^Nb^ALFA-L-mNG expressed without the presence of an ALFA tag and (ii) L-mNG without ^Nb^ALFA expressed in the presence of Shs1^ALFA^. (e) Images of ALFA-tagged proteins. From left to right (in each row) are the ALFA-tagged POI along with ^Nb^ALFA-L-mNG under the control of *P_TEF1_* (green), the mCherry-tagged marker protein or another location marker (red), and the merged image. First row, Myo1, a nonmuscle type II myosin, with Cdc3-mCherry as a bud neck marker; second row, Bud4, an anillin-like protein, with Cdc3-mCherry as a bud neck marker; third row, Dnm1, a mitochondrial fission protein, with Om45-mCherry as a mitochondrial marker; fourth row, Vph1, a vacuolar ATPase complex component, with FM4-64 dye (catalog no. T3166; Invitrogen) (20 μM final concentration) staining the vacuolar membrane. All of the strains were grown in synthetic complete (SC) medium at 30°C. Bar, 5 μm. Download FIG S1, PDF file, 1.7 MB. Copyright © 2022 Akhuli et al. 2022 Akhuli et al. https://creativecommons.org/licenses/by/4.0/ This content is distributed under the terms of the Creative Commons Attribution 4.0 International license.

An ideal live-cell imaging setup demands a high signal-to-noise ratio (SNR) at low laser excitation power to reduce phototoxicity while maintaining nanobody expression at a level such that the normal functioning of the tagged protein is not hampered. Thus, to optimize ^Nb^ALFA expression for quantitative live-cell imaging, we integrated detection plasmids expressing the ^Nb^ALFA-L-mNG construct under the control of different promoters into the strain carrying Shs1^ALFA^ (a terminal septin involved in cytokinesis [21]) and compared the fluorescence signal strengths across the different promoters using laser point scanning confocal microscopy (Fig. 2b). Shs1 was chosen due to its exclusive localization at the bud neck, which allows easy quantitative analysis across the range of promoters. A qualitative comparison of the relative signal strengths was first made at the same laser excitation power. We found that a strong fluorescence signal is achieved with nanobody expression under the control of the *P_GPD_* and *P_TEF1_* promoters, accompanied by low cytoplasmic background, while in the case of *P_ADH1_* and *P_CYC1_* promoters, the fluorescence signal is weaker (Fig. 2b). For a quantitative comparison, we analyzed the intensity profiles of each promoter across a line scan parallel to the mother-bud axis. We found large, clear peaks in the fluorescence intensity for *P_GPD_* and *P_TEF1_*, while the intensity peaks for *P_ADH1_* and *P_CYC1_* were comparable to the baseline levels (Fig. 2b; Fig. S1c). This set of observations implies that both *P_GPD_* and *P_TEF1_* offer an excellent signal-to-noise ratio compared to *P_ADH1_* and *P_CYC1_*. For *P_TEF1_* and *P_GPD_*, the fluorescence signal was specific, with minimal background, even at low laser power, making them ideal candidates for use in live-cell imaging. Control strains expressing mNeonGreen without the ALFA tag or ^Nb^ALFA expectedly showed a uniform cytoplasmic signal, lacking any foci corresponding to a localized protein (Fig. S1d). We therefore chose the *P_TEF1_* promoter for nanobody expression in all subsequent experiments. Next, we imaged proteins localizing to various subcellular locations using ALIBY, such as Shs1 (terminal septin) (21), Spc42 (component of the spindle pole body, the centrosome homolog in yeast) (22), Emc1 (endoplasmic reticulum [ER] membrane protein) (23), Om45 (mitochondrial outer membrane protein) (24), Exo84 (exocyst complex protein) (20), Myo1 (nonmuscle type II myosin) (25), Bud4 (anillin-like protein) (19), Dnm1 (mitochondrial fission protein) (26), and Vph1 (subunit of the vacuolar ATPase complex) (27), along with the appropriate subcellular location markers (Cdc3-mCherry [28], mCherry-Tub1 [29], Elo3-3mCherry [30], Om45-mCherry [24], and Cox4-mCherry [31]) (Fig. 2c; Fig. S1e). We obtained a similarly high signal-to-noise ratio without compromising protein localization while also preserving normal cellular morphology, confirming the utility of ALIBY for live-cell imaging of various yeast proteins.

### Live-cell imaging of ALFA-tagged proteins.

Next, to test the employability of ALIBY for live-cell imaging over extended durations, we proceeded to image ALFA-tagged proteins of various abundances that localize to various subcellular locations, such as the bud neck, exocyst, spindle pole body, nucleus, mitochondria, and vacuole, using the ^Nb^ALFA-L-mNG construct expressed under the control of the *P_TEF1_* promoter.

**FIG 3:**
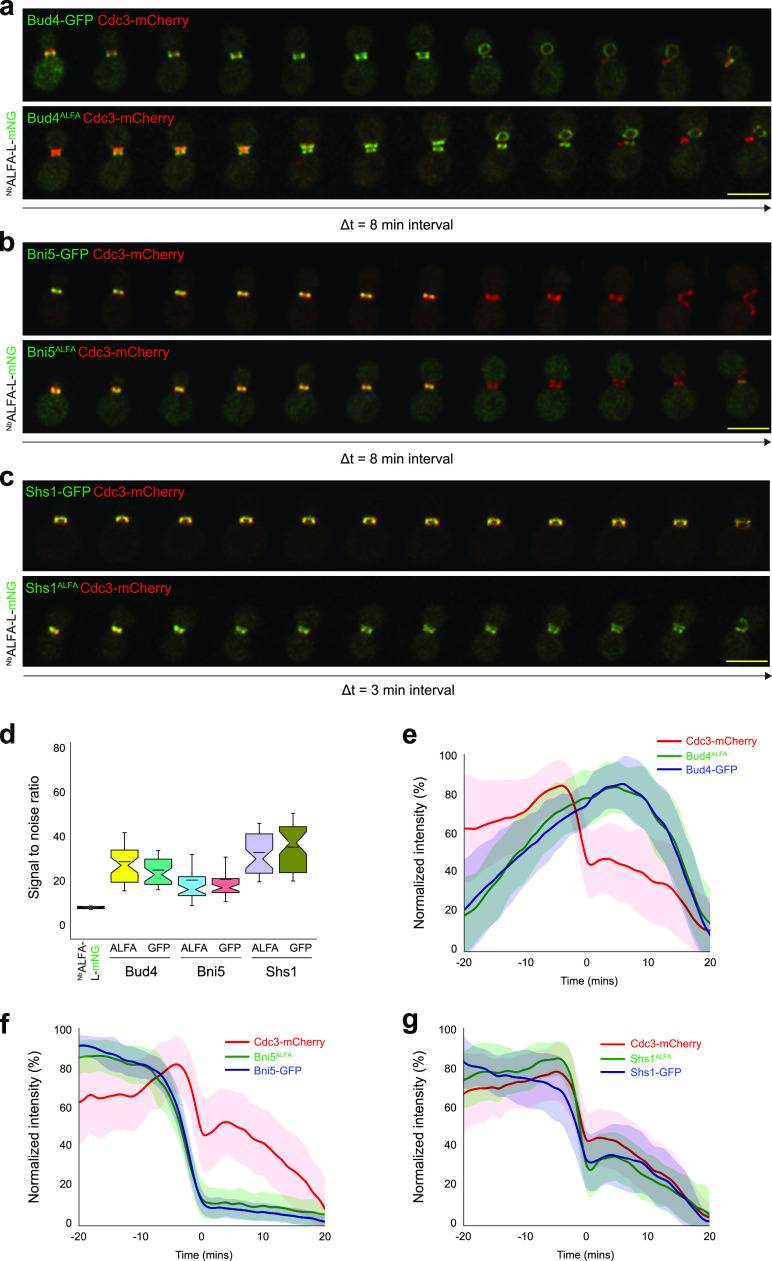
Time-lapse imaging and quantification of protein kinetics. The time interval between successive images shown in each montage is denoted as Δ*t*. (a) Bud4-GFP (top) and Bud4^ALFA^ (bottom) (Δ*t* = 8 min). (b) Bni5-GFP (top) and Bni5^ALFA^ (bottom) (Δ*t* = 8 min). (c) Shs1-GFP (top) and Shs1^ALFA^ (bottom) (Δ*t* = 3 min). (d) Signal-to-noise ratio comparison between C-terminal ALFA tag and GFP fusions of Bni5, Bud4, and Shs1 (*n* = 3 replicates; the number of cells quantified was 36 to 42 per strain). (e) Comparison of the kinetics of Bud4-GFP and Bud4^ALFA^ (*n* = 3 replicates; the number of cells quantified was 49 to 53 per strain). (f) Comparison of the kinetics of Bni5-GFP and Bni5^ALFA^ (*n* = 3 replicates; the number of cells quantified was 49 to 53 per strain). (g) Comparison of the kinetics of Shs1-GFP and Shs1^ALFA^ (*n* = 3 replicates; the number of cells quantified was 49 to 53 per strain). In addition to the ALFA-tagged proteins, the above-mentioned ALFA tag-containing strains also expressed ^Nb^ALFA-L-mNG under the control of *P_TEF1_*. All of the strains were grown in SC medium at 30°C. Green, ALFA-tagged proteins bound to ^Nb^ALFA-L-mNG and GFP-tagged proteins; red, Cdc3-mCherry. All of the time-lapse images were maximum-intensity projected for representation in montages. Bar, 5 μm.

To visualize the bud neck region and as a cell cycle marker, we used Cdc3-mCherry (28). We first imaged Bud4 (Fig. 3a; Videos S1 and S2), an anillin-like protein that marks the new bud site (19) and associates closely with septins at the bud neck. It is also known to regulate the septin double-ring structure (32). We then quantitatively compared the signal-to-noise ratio of Bud4^ALFA^ with that of conventionally C-terminally green fluorescent protein (GFP)-tagged Bud4 (Fig. 3d). We also find that the spatiotemporal localization pattern of Bud4^ALFA^ matches that of Bud4-GFP exactly, as depicted by their kinetic profiles at the bud neck (Fig. 3e), implying that the cell cycle kinetics of Bud4 remain unperturbed upon ALFA tagging compared to GFP tagging. We then observed Bni5, which interacts with and provides stability to the septin ring at the mother-bud neck (Fig. 3b; Videos S3 and S4) (33). Bni5 is recruited to the bud neck at the early bud stage and leaves just before the septin hourglass-to-double-ring (HDR) transition (34). The signal-to-noise ratio of Bni5^ALFA^ and its kinetics again closely match those of Bni5-GFP (Fig. 3d and f). Next, we visualized Shs1 (Fig. 3c), a terminal septin that is a member of the septin scaffold at the bud neck (21), which is essential for actomyosin ring assembly and disassembly (35). Shs1 is also known to recruit Bni5 to the mother-bud neck (36). We compared Shs1^ALFA^ and Shs1-GFP, and they also displayed similar signal-to-noise ratios and spatiotemporal kinetics at the bud neck (Fig. 3d and g), which strengthens the finding that the localized accumulation kinetics of the above-described proteins during the cell cycle are preserved in the ALFA-tagged strains, without any detectable off-target effects.

10.1128/msphere.00333-22.5 VIDEO S1 (Left) Bud4-GFP; (middle) Cdc3-mCherry; (right) merge (Δ*t* = 1 min). Bar, 5 μm. Download Movie S1, AVI file, 0.6 MB. Copyright © 2022 Akhuli et al. 2022 Akhuli et al. https://creativecommons.org/licenses/by/4.0/ This content is distributed under the terms of the Creative Commons Attribution 4.0 International license.

10.1128/msphere.00333-22.6 VIDEO S2 (Left) Bud4^ALFA^; (middle) Cdc3-mCherry; (right) merge (Δ*t* = 1 min). Bar, 5 μm. Download Movie S2, AVI file, 0.6 MB. Copyright © 2022 Akhuli et al. 2022 Akhuli et al. https://creativecommons.org/licenses/by/4.0/ This content is distributed under the terms of the Creative Commons Attribution 4.0 International license.

10.1128/msphere.00333-22.7 VIDEO S3 (Left) Bni5-GFP; (middle) Cdc3-mCherry; (right) merge (Δ*t* = 1 min). Bar, 5 μm. Download Movie S3, AVI file, 0.9 MB. Copyright © 2022 Akhuli et al. 2022 Akhuli et al. https://creativecommons.org/licenses/by/4.0/ This content is distributed under the terms of the Creative Commons Attribution 4.0 International license.

10.1128/msphere.00333-22.8 VIDEO S4 (Left) Bni5^ALFA^; (middle) Cdc3-mCherry; (right) merge (Δ*t* = 1 min). Bar, 5 μm. Download Movie S4, AVI file, 0.7 MB. Copyright © 2022 Akhuli et al. 2022 Akhuli et al. https://creativecommons.org/licenses/by/4.0/ This content is distributed under the terms of the Creative Commons Attribution 4.0 International license.

Next, we imaged proteins with diverse subcellular localizations, starting with Gin4, which is a protein kinase that coordinates septin ring assembly at the bud neck and promotes isotropic bud growth (Fig. S2a) (18). We then looked at Myo1, a type II myosin heavy chain protein that contributes to the generation of force required for cytokinesis and is brought to the early bud site by Bni5 (Fig. S2b and Video S5) (33). Myo1 associates with F-actin throughout anaphase to give rise to the actomyosin ring, which constricts to separate the daughter cell from the mother cell (37). Next, we imaged Hof1, an F-BAR protein associated with the septin scaffold at the bud neck, which organizes actin filaments and is part of the ingression-progression complex that coordinates primary septum formation with actomyosin ring constriction (Fig. S2c) (38). We then observed Cdc14, a phosphatase localized to the nucleolus (marked by Nab2-mCherry [39]), which is released during anaphase through the mitotic exit network (MEN) and Cdc fourteen early anaphase release (FEAR) pathways (Fig. S2d) (40, 41). Next, we visualized Spc42, a constituent of the spindle pole body. The spindle pole body duplicates in G_1_/S phase and migrates to opposite ends of the mother nuclear envelope forming the mitotic spindle (marked by mCherry-Tub1 [29]), and following cytokinesis, a copy of the spindle pole body moves to the daughter cell (Fig. S2e) (22). We then focused on visualizing proteins localizing to other subcellular locations, such as the exocyst, mitochondria, and vacuole, starting with Exo84, an important member of the exocyst complex involved in plasma membrane expansion and fusion through the delivery of secretory vesicles to active sites of exocytosis. In nascent buds, Exo84 concentrates at the bud tip, after which it gradually diffuses, and during cytokinesis, it shifts its localization to the bud neck (Fig. S2f and Video S6) (20). We continued with Om45, a mitochondrial outer membrane protein that is involved in porin channel regulation (Fig. S2g) (24, 42). Finally, we looked at Vph1, a subunit of the vacuolar membrane ATPase V_0_ domain involved in its assembly (Fig. S2h) (27).

10.1128/msphere.00333-22.2 FIG S2 Time-lapse imaging and spot assays. The time interval between successive images shown in each montage is denoted as Δ*t*. (a) Gin4^ALFA^ (Δ*t* = 6 min). (b) Myo1^ALFA^ (Δ*t* = 1 min). (c) Hof1^ALFA^ (Δ*t* = 4 min). (d) Cdc14^ALFA^ (Δ*t* = 3 min). (e) Spc42^ALFA^ (Δ*t* = 2 min). (f) Exo84^ALFA^ (Δ*t* = 6 min). (g) Om45^ALFA^ (Δ*t* = 3 min). (h) Vph1^ALFA^ (Δ*t* = 3 min). In addition to the ALFA-tagged proteins, the above-mentioned strains also expressed ^Nb^ALFA-L-mNG under the control of *P_TEF1_*. All of the strains were grown in synthetic complete (SC) medium at 30°C. Green, ALFA-tagged proteins bound to ^Nb^ALFA-L-mNG; red, mCherry-tagged marker proteins (Cdc3 for panels a to c and f, Nab2 for panel d, Tub1 for panel e, and Cox4 for panel g). All of the time-lapse images were maximum-intensity projected for representation in montages. Bar, 5 μm. (i) Comparison of the growth rates of strains containing POI^ALFA^ with those of the wild-type strain, control strains, and POI-GFP-containing strains. Five serial dilutions for each strain were spotted onto YPD plates, which were grown at 23°C, 30°C, and 37°C. Download FIG S2, PDF file, 2.1 MB. Copyright © 2022 Akhuli et al. 2022 Akhuli et al. https://creativecommons.org/licenses/by/4.0/ This content is distributed under the terms of the Creative Commons Attribution 4.0 International license.

All of the ALFA-tagged strains exhibited normal cell morphologies in our time-lapse studies and showed growth rates similar to those of the wild-type strain and the strains containing their GFP-tagged counterparts in a spot assay (Fig. S2i). We also find that the presence of the tag or the nanobody in isolation or in combination does not affect cellular growth across a wide range of temperatures. Furthermore, as discussed above, we observe that the cell cycle kinetics across diverse proteins remain preserved upon fusion with a C-terminal ALFA tag. Thus, in summary, ALIBY offers an excellent signal-to-noise ratio for long-duration quantitative live-cell imaging of a variety of differentially abundant yeast proteins while preserving normal cell physiology, growth rates, protein localization, and kinetics.

## DISCUSSION

The ALFA tag was designed and characterized as a competent, all-in-one tag with a unique sequence absent in commonly used eukaryotic model organisms, which can be reliably used to study protein functions (3). This 13-amino-acid tag possesses many desirable features, such as versatility, compactness, high solubility, and the ability to refold upon stringent chemical treatments (3), which underscores the tag’s suitability for diverse applications such as live-cell imaging, immunoblotting, and immunoprecipitation, making the ALFA/^Nb^ALFA system a powerful tool for studying protein dynamics and function.

Here, we have extended the benefits of the ALFA/^Nb^ALFA system to yeast research through a toolkit comprising a series of vectors specifically designed for tagging and detecting any POI. These vectors are ready to use, requiring no further cloning steps, with several selectable markers to choose from, to introduce the ALFA tag or ^Nb^ALFA-L-mNG into the strain of interest. We have based our tagging plasmids on the well-established pYM series of vectors, which rely on the chromosomal integration of PCR-amplified cassettes by homologous recombination (1). This makes the adaptability of ALIBY into the conventional yeast genetics workflow seamless and convenient, as the widely used long C-terminal tagging primers (13) (S2/S3) are compatible with our kit. Similarly, the detection plasmids build upon the commonly used pRS30 series of vectors and can be readily integrated into the genome after linearization with the same enzymes as the ones used for the pRS series.

We have demonstrated an extensive set of applications of ALIBY through live-cell imaging and biochemical studies of various yeast proteins. Through our primary focus on live-cell imaging of numerous proteins localized at various subcellular locations, we have established the toolkit’s ability to derive an excellent signal-to-noise ratio with no detectable off-target effects for all proteins tested in our study at different subcellular locations, thus making it suitable for quantitative imaging over an extended time without affecting cellular growth and morphology. In addition, imaging of conventional fluorescent-protein-tagged POIs over long periods of time inevitably brings with it the drawback of a loss of signal intensity due to photobleaching. ALIBY might not suffer from this drawback since the high on- and off-rates of the ALFA/^Nb^ALFA complex (3) imply that ^Nb^ALFA-L-mNG molecules from the diffuse cytoplasmic pool can replace the POI^ALFA^-bound pool. This might effectively compensate for the photobleaching that takes place at the specific POI location during longer durations of live-cell imaging. ALIBY can also be employed for biochemical and cell biology applications such as superresolution microscopy, coimmunoprecipitation, and mass spectrometry, making it a valuable asset for the yeast research community. By simplifying the workflow for yeast protein investigations while employing the significant benefits of the ALFA tag over other epitope tags, we hope that ALIBY will contribute to the acceleration of yeast research and encourage innovative experimental designs to address a wider range of fundamental questions.

## MATERIALS AND METHODS

### Cloning and construction of plasmids.

All plasmids used in this study are listed in Table S1 in the supplemental material. For constructing the tagging plasmids, oligonucleotides carrying the ALFA tag sequence codon optimized for yeast and flanked by SalI and BglII sites were synthesized by Sigma-Aldrich. These oligonucleotides were annealed in annealing buffer (10 mM Tris [pH 7.5 to 8.0], 50 mM NaCl, 1 mM EDTA) as recommended by Addgene (https://www.addgene.org/protocols/annealed-oligo-cloning) and then digested with SalI and BglII, followed by ligation with SalI-BglII-digested pYM14/16/17 and pFA6a-*HIS3MX6* (which contains the *his5* gene from Schizosaccharomyces pombe [43]), to give rise to the tagging plasmids piSP532, piSP534, piSP536, and piSP538.

10.1128/msphere.00333-22.3 TABLE S1 Plasmids used in this study. Download Table S1, PDF file, 0.03 MB. Copyright © 2022 Akhuli et al. 2022 Akhuli et al. https://creativecommons.org/licenses/by/4.0/ This content is distributed under the terms of the Creative Commons Attribution 4.0 International license.

For constructing the detection plasmids, a sequential strategy was adopted. Promoter–multiple-cloning site (MCS)–*term_CYC1_* terminator of the CYC1 gene from saccharomyces cerevisiae fragments were PCR amplified from the pRS414/pRS415 vector carrying the *P_CYC1_*/*P_ADH1_*/*P_GPD_*/*P_TEF1_* promoter. These fragments were cloned into pRS305 (linearized with XbaI and XhoI) using NEBuilder HiFi DNA assembly according to the manufacturer’s instructions (catalog no. E2621; New England BioLabs). They were subsequently transformed into Escherichia coli TOP10 cells, and plasmids were isolated using a QIAprep spin miniprep kit (catalog no. 27106; Qiagen) and confirmed by restriction digestion. The ^Nb^ALFA-L-mNG sequence was synthesized and cloned as mentioned above into the above-described vectors (linearized with BamHI) to give rise to piSP551, piSP553, piSP555, and piSP557, respectively, which are our detection plasmids. Based on the imaging results, the ^Nb^ALFA-L-mNG construct along with *P_TEF1_* and *term_CYC1_* were then cloned as described above into pRS303, pRS304, and pRS306 (linearized with BamHI) to generate piSP765, piSP767, and piSP565, respectively, which are the rest of our detection plasmids. For constructing the control plasmid piSP763, the L-mNG fragment was amplified from piSP557 and cloned into BamHI-digested piSP525. piSP629 was constructed by cloning mCherry (amplified from mCherry-pBAD [catalog no. 54630; Addgene]) into pYM17 (digested with SalI and BglII). All of the above-described plasmids were confirmed by insert release using restriction digestion and sequencing and will be submitted to Addgene.

### Construction of yeast strains.

All yeast strains constructed for this study are listed in Table S2. The wild-type strain used was Saccharomyces cerevisiae ESM356, derived from the S288C genetic background. Yeast strains were cultured at 30°C with rotation at 180 rpm (44). For yeast strain construction, we used yeast extract-peptone-dextrose (YPD) agar plates supplemented with antibiotics, including 100 μg/mL G418 (catalog no. 58327; Sisco Research Laboratories Pvt. Ltd.), 100 μg/mL nourseothricin (catalog no. AB-102; Jena Bioscience), and 50 μg/mL hygromycin B (catalog no. 67317; Sisco Research Laboratories Pvt. Ltd.), or synthetic complete (SC) medium agar plates lacking leucine, uracil, or tryptophan and the corresponding liquid media.

10.1128/msphere.00333-22.4 TABLE S2 Yeast strains used in this study. Download Table S2, PDF file, 0.03 MB. Copyright © 2022 Akhuli et al. 2022 Akhuli et al. https://creativecommons.org/licenses/by/4.0/ This content is distributed under the terms of the Creative Commons Attribution 4.0 International license.

In order to introduce the bud neck marker (pMO014) (Cdc3-mCherry) and the tubulin marker (pAK011) (mCherry-Tub1), the corresponding plasmids were linearized with BglII and NotI, respectively (catalog no. R0144 and R3189; New England BioLabs) and transformed for integration into the genomic *trp1* and *ura3* loci, respectively, using a lithium acetate (LiOAc)-based protocol (14). To introduce the organelle and cytoplasmic markers, PCR amplification was done from plasmid piSP629 using the S2/S3 primers for Cox4, Om45, and Nab2, and from plasmid pMAM12-1 for Elo3; the linear fragments were then transformed to obtain C-terminal mCherry fusions of these proteins. For fusing the ALFA tag to the C termini of the desired set of proteins, PCR amplification was done from piSP534 using the appropriate S2/S3 primers, and the linear fragments were transformed into the appropriate strains containing the corresponding location markers. For the integration of C-terminally mNeonGreen-fused ^Nb^ALFA into the genomic *leu2* locus, piSP522, piSP523, piSP524, and piSP525 were linearized with KasI (catalog no. R0544; New England BioLabs) and transformed into the above-described strains as appropriate. To construct strain YSP210, plasmid piSP763 was linearized with KasI and transformed into the wild-type ESM356 strain YSP002. The PCR fragment for ALFA-tagged Shs1 generated using the S2/S3 primers was then transformed into YSP210 to give rise to strain YSP248. PCR-amplified fragments from pYM25 using S2/S3 primers for Bud4, Bni5, and Shs1 were transformed to obtain C-terminal GFP fusions in a strain carrying Cdc3-mCherry.

### Spot assay.

Yeast strains were precultured in YPD broth overnight at 30°C with rotation at 180 rpm and subcultured to a final optical density at 600 nm (OD_600_) of 1. Serial 10^0^, 10^−1^, 10^−2^, 10^−3^, and 10^−4^ dilutions were made in YPD, and 4 μL of each dilution was spotted onto 3 YPD agar plates. The plates were incubated at 23°C, 30°C, and 37°C, respectively, for 48 h, after which the plates were scanned.

### Immunofluorescence.

Immunofluorescence (IF) analysis was performed using a modified version of a protocol described previously (45). Briefly, yeast strains were grown in YPD broth at 25°C overnight. The culture grown overnight was diluted to a starting OD_600_ of 0.1 and allowed to grow at 25°C until mid-log phase was reached (OD_600_ = 0.4 to 0.6). Cells were fixed with 4% formaldehyde for 60 min at 25°C. The cells were then pelleted at 750 × *g* for 5 min, washed three times with 1× phosphate-buffered saline (PBS), and finally resuspended in 200 μL of 1.2 M sorbitol-phosphate-citrate (SPC) buffer (1.2 M sorbitol, 1 M K_2_HPO_4_, 1 M citric acid). To digest the cell wall, 25 μL of Long-Life Zymolase (catalog no. 786-036; G-Biosciences) was added, and the suspension was incubated with mild shaking at 37°C for 60 min. The digested cells were then pelleted by centrifugation at 750 × *g* for 5 min, washed with ice-cold SPC buffer three times, and finally incubated with 500 μL of blocking buffer at 25°C for 15 min with shaking (2% bovine serum albumin [BSA] plus 0.1% Triton X-100 in PBS). The cells were again pelleted and resuspended in 500 μL of antibody dilution buffer (1% BSA plus 0.05% Triton X-100 in PBS) containing FluoTag-X2 anti-ALFA^Alexa Fluor 647^ (catalog no. N1502-AF647-L; NanoTag Biotechnologies, Germany) at a final dilution of 1:500. The cells were then incubated overnight with rotation at 4°C. The next day, the cells were pelleted, washed three times with 1× PBS, and finally resuspended in 20 μL of 1× PBS. One microliter of the final suspension was mounted onto a glass slide, and a cover glass was placed on top. Images were taken using a fully motorized Olympus IX83 microscope (Olympus Life Science, Japan) (100× oil objective, 1.45 numerical aperture [NA]) equipped with a pE-4000 LED-based light source (CoolLED Ltd., UK) and a Prime BSI scientific CMOS (complementary metal oxide semiconductor) camera (Teledyne Photometrics, USA). All images were processed in Fiji for representation (Fig. S1b).

### Live-cell imaging.

For tethering yeast cells to glass-bottom dishes (35 mm, catalog no. 81218-200; ibidi GmbH) for live-cell imaging, we coated the dishes with 6% concanavalin A type 6 (catalog no. C2010; Sigma-Aldrich). Yeast cells were precultured overnight, subcultured for 3 to 4 h to mid-log phase (OD_600_ = 0.5 to 0.8) in SC broth at 30°C with rotation at 180 rpm, and plated onto concanavalin A-coated dishes. Live-cell imaging was performed using an Olympus FV3000 laser point scanning confocal microscope equipped with a 100× oil objective, high-sensitivity GaAsP photomultiplier tube (PMT) detectors, and solid-state lasers (488 nm and 561 nm). Images were acquired using Olympus FluoView 3000 (2.4.1.198) software. All time-lapse movies consisted of 7 z-slices at a step size of 0.5 μm for a period of 60 to 180 min with a 1-min interval. Three-dimensional (3D) deconvolution of raw images was done using Olympus CellSens Dimension (3.1) software, followed by maximum-intensity projection using Fiji for representation in a montage. All images in Fig. 2c and Fig. S1e were acquired at a 2,048-by-2,048 resolution under the conditions described above.

### Image analysis and quantification.

All microscopy data were quantified using Fiji (ImageJ2). The raw image data were sum-intensity projected, and protein kinetics at the region of interest (ROI) were analyzed using the quantification workflow described previously (46). In brief, raw data were registered using the ImageJ plug-in StackReg. A polygon (ROI) was drawn to encompass the fluorescence signal at the bud neck region, and the derived intensities were corrected by background subtraction. The quantification data were extracted, normalized, and further analyzed to derive the temporal protein kinetics. The graphs representing kinetic signatures of individual proteins (Fig. 3e to g) were plotted using Origin (2015, Sr2, 69.2.272; OriginLab, USA).

The signal-to-noise ratio (SNR) was calculated on sum-intensity-projected time-lapse data analyzed in Fiji. SNR values were obtained using the following formula (47): SNR = (maximum intensity of signal − mean intensity of background signal)/standard deviation of background signal.

A notched box plot was used to represent the SNR values (Fig. 3d [the notched box graph represents 25% and 75% intervals, the black lines represent the means, the notches represent the medians, and the whiskers denote the standard deviations]).

### Immunoblotting.

Yeast cultures were grown in YPD broth at 30°C until mid-log phase was reached, and cell lysates were prepared using a trichloroacetic acid (TCA)-based protocol as described previously (13) or a glass bead lysis method (see the section on immunoprecipitation, below). To summarize, yeast cells corresponding to 2-mL cultures were pelleted at 20,000 × *g* (full speed) for 3 min, and after the removal of the supernatant, the cells were resuspended in 800 μL of cold water and vortexed. Next, they were incubated on ice with 150 μL of 1.85 M NaOH for 5 min, followed by incubation on ice with 150 μL of 55% TCA for 20 min. The lysates were then pelleted at full speed for 20 min, and the supernatant was removed. The pellet was resuspended in 75 μL of high urea dithiothreitol buffer (HU-DTT) (8 M urea, 5% [wt/vol] SDS, 200 mM Tris-Cl [pH 6.8], 0.1 mM EDTA, bromophenol blue, 15 mg/mL of DTT) and vortexed, followed by neutralization with 1 μL of 2 M Tris. The samples were then incubated at 95°C for 5 min and centrifuged at full speed for 2 min. A total of 10 to 30 μL of the supernatants (based on protein abundances) was run on an 8% SDS-PAGE gel and transferred to a nitrocellulose membrane (catalog no. 1620112; Bio-Rad) and an Immobilon 0.45-μm polyvinylidene difluoride (PVDF) membrane (catalog no. IPVH00010; Millipore) using a semidry transfer method (Bio-Rad) (at 0.11 Ampere limited to 16 V for 1.5 h) for immunoblotting. The membrane was blocked with 5% skimmed milk in 1× PBS-Tween (PBS-T) for 1 h at room temperature, followed by incubation with a 1:500 dilution of HRP-conjugated sdAb anti-ALFA primary antibody (catalog no. N1505, lot no. 15201101; NanoTag Biotechnologies) overnight at 4°C. The membrane was then washed three times with 1× PBS-T, and the blot was developed using the Immobilon Forte Western HRP substrate (catalog no. WBLUF0100; Millipore). Images were acquired using ImageQuant LAS500 (Cytiva, USA). The same blot was stripped with stripping buffer (0.2 M glycine, 0.1% [wt/vol] SDS, and 1% [vol/vol] Tween 20, with the pH adjusted to 2.2) for 30 min and blocked again. Immunoblotting was performed to detect actin and tubulin as loading controls using a 1:10,000 dilution of mouse antiactin monoclonal antibody (catalog no. MA1-744, lot no. WC317616; Invitrogen)/mouse anti-α-tubulin monoclonal antibody (catalog no. MA1-8001, lot no. WH3238708; Invitrogen) and a 1:20,000 dilution of HRP-conjugated rabbit anti-mouse secondary antibody (catalog no. 7076, lot no. 36; Cell Signaling Technology), and the blot was developed similarly.

### Immunoprecipitation.

Yeast cultures were grown in YPD broth at 30°C until log phase was reached (OD_600_ = 1), and total cell extracts (TCEs) were prepared as described previously (48), modified for our purpose. To summarize, yeast cells corresponding to 250-mL cultures were pelleted at 2,000 × *g* for 5 min (all centrifugation steps were performed at 4°C), and the pellets were washed with 1× PBS. After the removal of the supernatant, the pellet was resuspended in 2 mL of lysis buffer (49) (50 mM Tris-HCl [pH 7.5], 150 mM NaCl, 1 mM EDTA, 10% glycerol, 1 mM DTT, 1× phenylmethylsulfonyl fluoride [PMSF], 1× protease inhibitor cocktail [PIC]) and transferred to 2-mL conical FastPrep vials (4 vials, 500 μL each), to which an equal volume of chilled, acid-washed glass beads was added. Cell lysis was carried out at 4°C using a FastPrep-24 cell disruptor (catalog no. 6004500; MP Biomedicals) set at 6.5 for 8 cycles of 1 min each, with 2 min of incubation in an ice water bath between cycles. Lysis was verified using trypan blue staining. Caps of the FastPrep vials were perforated using a sterile needle, and they were inverted into 15-mL conical tubes. The lysate was collected by centrifugation at 1,000 × *g* for 1.5 min and transferred to a 1.5-mL microcentrifuge tube. The collected lysates were incubated on ice with 0.1% Triton X-100 before being clarified by centrifugation at 16,000 × *g* for 10 min. The clarification step was repeated twice, while collecting the supernatant at the end of each step. Upon clarification, the protein concentration was estimated using a standard Bradford assay (50). The rest of the clarified lysates (corresponding to 1 mg of total protein) was incubated with 50 μL of a washed ALFA Selector^ST^ bead slurry (catalog no. N1511-L; NanoTag Biotechnologies) at 4°C for 3 h with head-over-tail rotation. The beads were sedimented by centrifugation at 1,000 × *g* for 1.5 min, and the supernatant was removed. The beads were then washed three times with lysis buffer containing 0.1% Triton X-100. The TCE and bead samples were incubated with 5× SDS sample loading buffer (5% [wt/vol] SDS, 25% [vol/vol] glycerol, 1 M Tris-Cl [pH 6.8], 0.05% [wt/vol] bromophenol blue, 1% [vol/vol] β-mercaptoethanol) at 95°C for 10 min and then centrifuged at full speed for 2 min. The supernatants of TCE and bead samples were run on an 8% SDS-PAGE gel alongside a PAGEmark Tricolor Plus protein marker (catalog no. 786-419; G-Biosciences). Immunoblotting was performed using the protocol mentioned above.

10.1128/msphere.00333-22.9 VIDEO S5 (Left) Myo1^ALFA^; (middle) Cdc3-mCherry; (right) merge (Δ*t* = 1 min). Bar, 5 μm. Download Movie S5, AVI file, 0.3 MB. Copyright © 2022 Akhuli et al. 2022 Akhuli et al. https://creativecommons.org/licenses/by/4.0/ This content is distributed under the terms of the Creative Commons Attribution 4.0 International license.

10.1128/msphere.00333-22.10 VIDEO S6 (Left) Exo84^ALFA^; (middle) Cdc3-mCherry; (right) merge (Δ*t* = 1 min). Bar, 5 μm. Download Movie S6, AVI file, 0.5 MB. Copyright © 2022 Akhuli et al. 2022 Akhuli et al. https://creativecommons.org/licenses/by/4.0/ This content is distributed under the terms of the Creative Commons Attribution 4.0 International license.
