# Supplementary figures and images for: ALIBY: ALFA Nanobody-Based Toolkit for Imaging and Biochemistry in Yeast
Source: mSphere. 2022 Oct 3;7(5):e00333-22. doi: 10.1128/msphere.00333-22 (PMC9599267; doi:10.1128/msphere.00333-22)

# Figure S1

**a**

POI<sup>ALFA</sup>

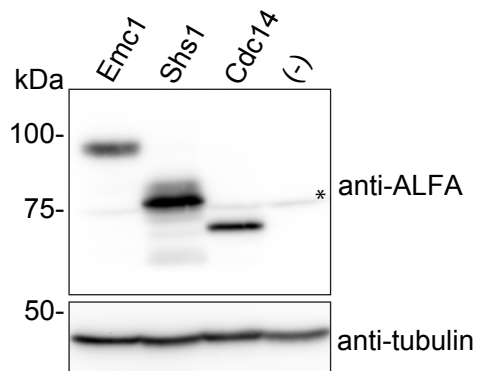

**c**

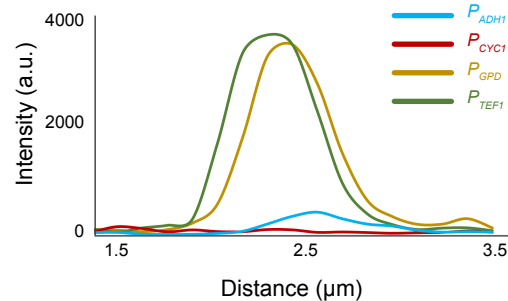

**d**

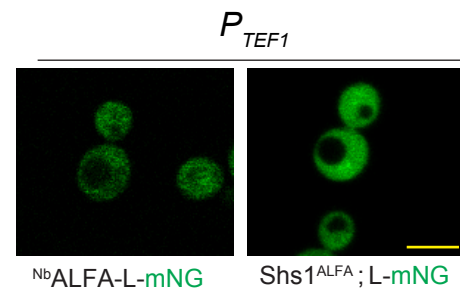

**b**

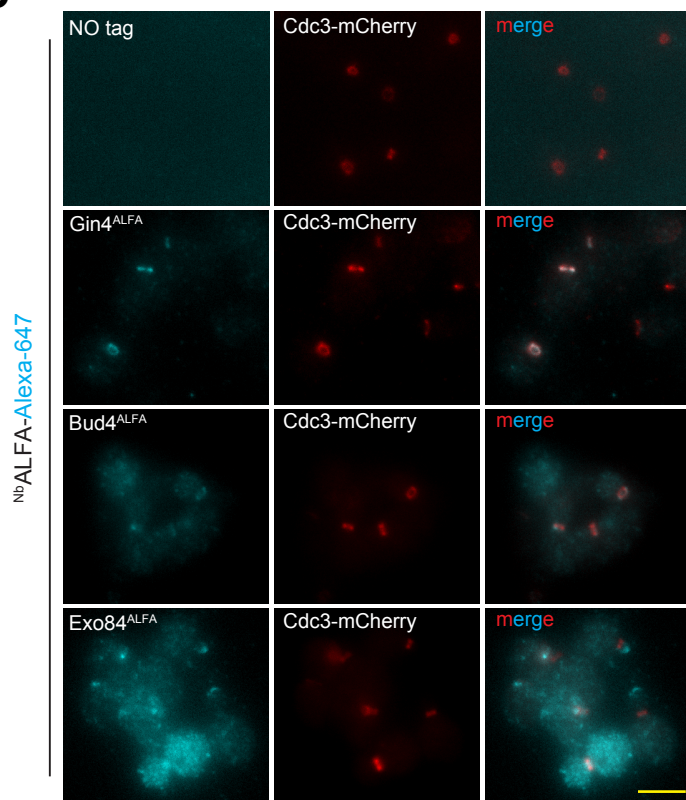

**e**

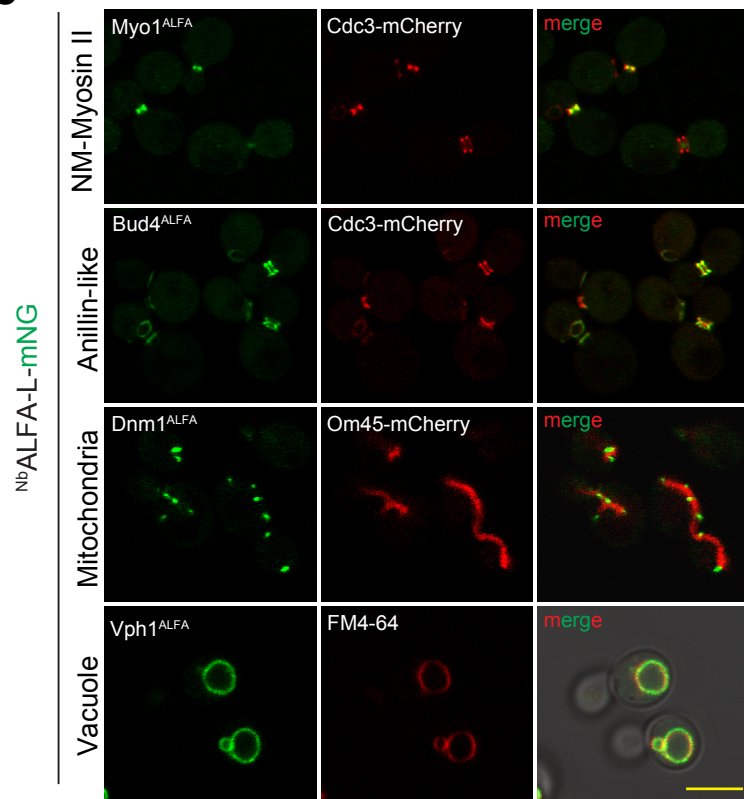

Supplement: FIG S1 [file msphere.00333-22-s0001.pdf]

Figure S2

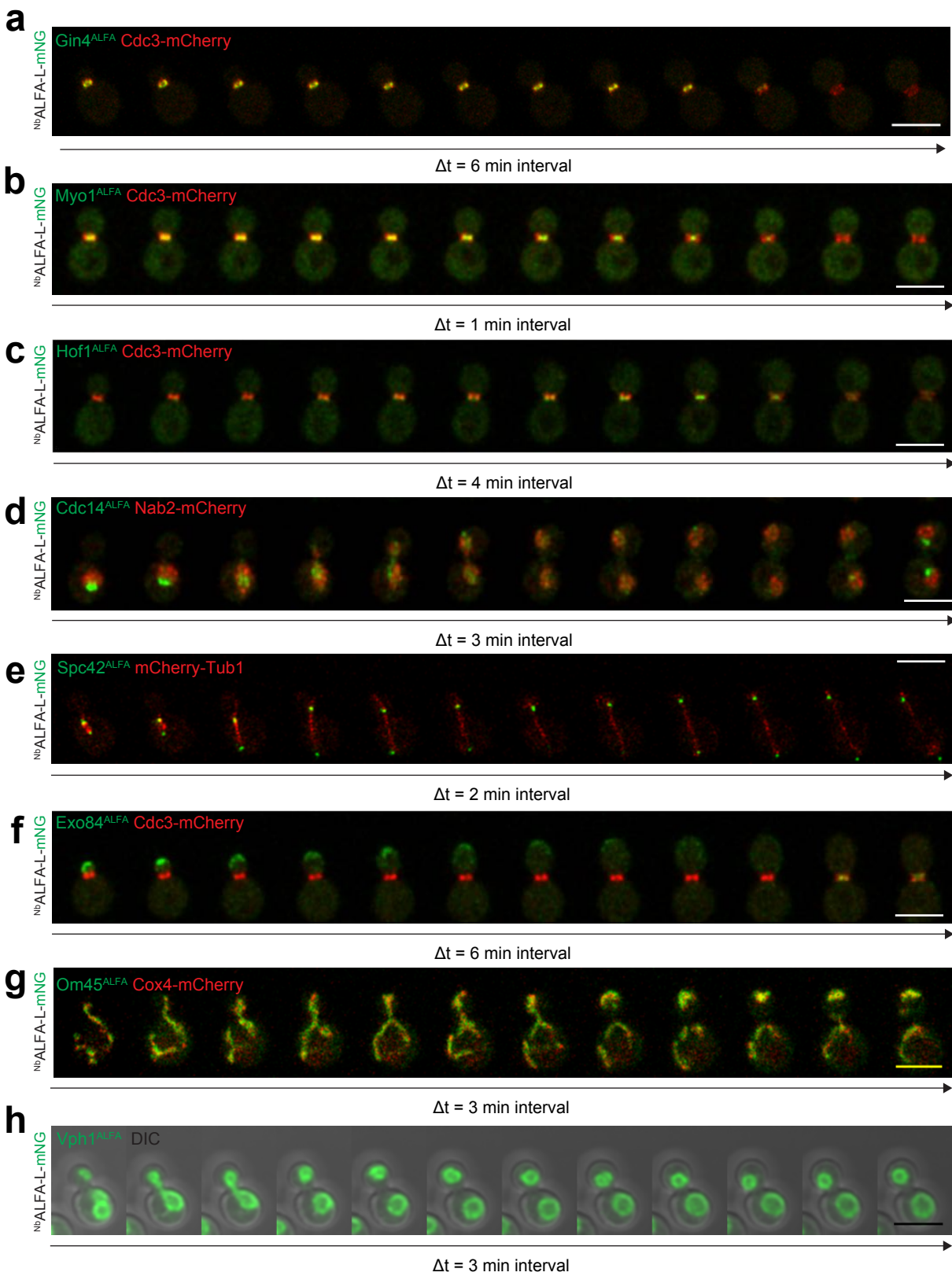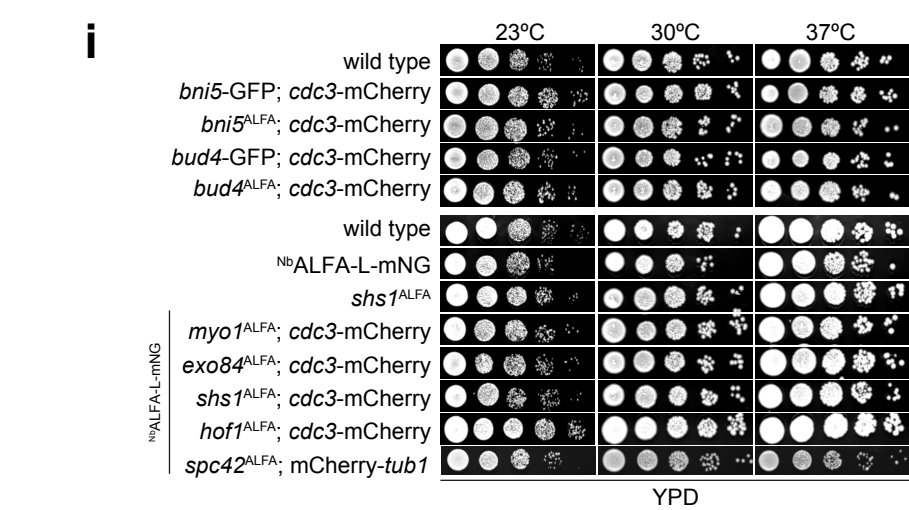

Supplement: FIG S2 [file msphere.00333-22-s0008.pdf]
