# Supplementary material for: ALIBY: ALFA Nanobody-Based Toolkit for Imaging and Biochemistry in Yeast
Source: mSphere. 2022 Oct 3;7(5):e00333-22. doi: 10.1128/msphere.00333-22 (PMC9599267; doi:10.1128/msphere.00333-22)
Supplement: TABLE S1 [file msphere.00333-22-s0009.pdf]

Supplementary Table 1: Plasmids used in this study

Plasmid No.	Plasmid Description	Reference
	pRS303	Sikorski and Hieter (1)
	pRS304	Sikorski and Hieter (1)
	pRS305	Sikorski and Hieter (1)
	pRS306	Sikorski and Hieter (1)
	pRS414-P_{CYC1}	Mumberg, Muller, Funk (2)
	pRS415-P_{ADH1}	Mumberg, Muller, Funk (2)
	pRS415-P_{GPD}	Mumberg, Muller, Funk (2)
	pRS415-P_{TEF1}	Mumberg, Muller, Funk (2)
	pYM14	Knop *et al.* (3)
	pYM16	Knop *et al.* (3)
	pYM17	Knop *et al.* (3)
	pYM25	Knop *et al.* (3)
	pFA6a-*HIS3MX6*	Knop *et al.* (3)
piSP532	pYM14-ALFA-*kanMX4*	This study
piSP534	pYM16-ALFA-*hphNT1*	This study
piSP536	pYM17-ALFA-*natNT2*	This study
piSP538	pFA6a-ALFA-*HIS3MX6*	This study
piSP551	pRS305-P_{ADH1}-^{Nb}ALFA-L-mNG-$term_{CYC1}$	This study
piSP553	pRS305-P_{CYC1}-^{Nb}ALFA-L-mNG-$term_{CYC1}$	This study
piSP555	pRS305-P_{GPD}-^{Nb}ALFA-L-mNG-$term_{CYC1}$	This study
piSP557	pRS305-P_{TEF1}-^{Nb}ALFA-L-mNG-$term_{CYC1}$	This study
piSP565	pRS306-P_{TEF1}-^{Nb}ALFA-L-mNG-$term_{CYC1}$	This study
piSP629	pYM17-mCherry-*natNT2*	This study
	pMAM12-1	From Prof. Gislene Pereira
	pAK011	Khmelinskii *et al.* (4)

piSP763	pRS305-P_{TEF1}-L-mNG-$term_{CYC1}$	This study
piSP765	pRS303-P_{TEF1}-^{Nb}ALFA-L-mNG-$term_{CYC1}$	This study
piSP767	pRS304-P_{TEF1}-^{Nb}ALFA-L-mNG-$term_{CYC1}$	This study
pMO014	YIp204-*cdc3*-mCherry-*TRP1*	Fang *et al.* (5)

References

1. Sikorski RS, Hieter P. 1989. A system of shuttle vectors and yeast host strains designed for efficient manipulation of DNA in Saccharomyces cerevisiae. Genetics 122:19–27.

2. Mumberg D, Müller R, Funk M. 1995. Yeast vectors for the controlled expression of heterologous proteins in different genetic backgrounds. Gene 156:119–122.

3. Knop M, Siegers K, Pereira G, Zachariae W, Winsor B, Nasmyth K, Schiebel E. 1999. Epitope Tagging of Yeast Genes using a PCR-based Strategy: More Tags and Improved Practical Routines. Yeast 15:963–972.

4. Khmelinskii A, Lawrence C, Roostalu J, Schiebel E. 2007. Cdc14-regulated midzone assembly controls anaphase B. Journal of Cell Biology 177:981–993.

5. Fang X, Luo J, Nishihama R, Wloka C, Dravis C, Travaglia M, Iwase M, Vallen EA, Bi E. 2010. Biphasic targeting and cleavage furrow ingression directed by the tail of a myosin II. J Cell Biol 191:1333.
