# Supplementary material for: ALIBY: ALFA Nanobody-Based Toolkit for Imaging and Biochemistry in Yeast
Source: mSphere. 2022 Oct 3;7(5):e00333-22. doi: 10.1128/msphere.00333-22 (PMC9599267; doi:10.1128/msphere.00333-22)
Supplement: TABLE S2 [file msphere.00333-22-s0010.pdf]

Supplementary Table 2: Yeast strains used in this study

**Strain No.**	**Description**
YSP002	ESM356 (*MAT***a** *ura3*-52 *leu2*Δ1 *trp1*Δ63 *his3*Δ200) WT
YSP126	YSP002 *trp1*$^{+}$:*cdc3*-mCherry *bud4*-GFP::*hphNT1*
YSP128	YSP002 *leu2*$^{+}$:pRS305-P_{ADH1}-^{Nb}ALFA-L-mNG-$term_{CYC1}$
YSP130	YSP002 *leu2*$^{+}$:pRS305-P_{CYC1}-^{Nb}ALFA-L-mNG-$term_{CYC1}$
YSP132	YSP002 *leu2*$^{+}$:pRS305-P_{GPD}-^{Nb}ALFA-L-mNG-$term_{CYC1}$
YSP134	YSP002 *leu2*$^{+}$:pRS305-P_{TEF1}-^{Nb}ALFA-L-mNG-$term_{CYC1}$
YSP135	YSP134 *trp1*$^{+}$:*cdc3*-mCherry *bud4*-ALFA::*hphNT1*
YSP136	YSP134 *trp1*$^{+}$:*cdc3*-mCherry *myo1*-ALFA::*hphNT1*
YSP137	YSP134 *trp1*$^{+}$:*cdc3*-mCherry *exo84*-ALFA::*hphNT1*
YSP138	YSP134 *trp1*$^{+}$:*cdc3*-mCherry
YSP157	YSP134 *dnm1*-ALFA::*hphNT1*
YSP159	YSP134 *spc42*-ALFA::*hphNT1*
YSP160	YSP134 *cox4*-mCherry::*natNT2*
YSP167	YSP134 *elo3*-3mCherry::*kanMX6*
YSP169	YSP203 *cdc14*-ALFA::*hphNT1*
YSP171	YSP134 *trp1*$^{+}$:*cdc3*-mCherry *shs1*-ALFA::*hphNT1*
YSP172	YSP134 *trp1*$^{+}$:*cdc3*-mCherry *bni5*-ALFA::*hphNT1*
YSP173	YSP167 *emc1*-ALFA::*hphNT1*
YSP201	YSP134 *trp1*$^{+}$:*cdc3*-mCherry *hof1*-ALFA::*hphNT1*
YSP203	YSP134 *nab2*-mCherry::*natNT2*
YSP210	YSP002 *leu2*$^{+}$:pRS305-P_{TEF1}-L-mNG-$term_{CYC1}$ *trp1*$^{+}$:*cdc3*-mCherry
YSP248	YSP210 *shs1*-ALFA::*hphNT1*
YSP286	YSP002 *trp1*$^{+}$:*cdc3*-mCherry *bni5*-GFP::*hphNT1*
YSP287	YSP002 *trp1*$^{+}$:*cdc3*-mCherry *shs1*-GFP::*hphNT1*
YSP299	YSP002 *gin4*-ALFA::*hphNT1*

YSP386	YSP159 *ura3*$^{+}$:mCherry-*tub1*
YSP395	YSP002 *bud4*-ALFA::*hphNT1*
YSP398	YSP299 *his3*$^{+}$:*shs1*-9Myc
YSP405	YSP002 *his3*$^{+}$:*shs1*-9Myc
YSP462	YSP134 *vph1*-ALFA::*hphNT1*
YSP463	YSP299 *trp1*$^{+}$:*cdc3*-mCherry
YSP464	YSP395 *trp1*$^{+}$:*cdc3*-mCherry
YSP465	YSP134 *gin4*-ALFA::*hphNT1*
YSP466	YSP465 *trp1*$^{+}$:*cdc3*-mCherry
YSP467	YSP002 *exo84*-ALFA::*hphNT1*
YSP497	YSP160 *om45*-ALFA::*hphNT1*
YSP498	YSP157 *om45*-mCherry::*natNT2*
